# Fabrication of an Integrated, Flexible, Wireless Pressure Sensor Array for the Monitoring of Ventricular Pressure

**DOI:** 10.3390/mi15121435

**Published:** 2024-11-28

**Authors:** Natiely Hernández-Sebastián, Daniela Diaz-Alonso, Bernardino Barrientos-García, Francisco Javier Renero-Carrillo, Wilfrido Calleja-Arriaga

**Affiliations:** 1 Centro de Investigaciones en Óptica, A.C., CIO, Leon 37150, Mexico; bb@cio.mx; 2 CD-MEMS INAOE, Puebla 72840, Mexico; dandiaz488@gmail.com; 3 Instituto Nacional de Astrofísica, Óptica y Electrónica—INAOE, Puebla 72840, Mexico; paco@inaoep.mx

**Keywords:** capacitive pressure sensor, flexible electronics, passive wireless system, ventricular pressure monitoring, implantable BioMEMS

## Abstract

This work presents the design, fabrication, and rigorous validation of a flexible, wireless, capacitive pressure sensor for the full-range continuous monitoring of ventricular pressure. The proposed system consists of an implantable set and an external readout device; both modules were designed to form an RCL resonant circuit for passive, wireless pressure sensing and signal retrieving. Using surface micromachining and flexible electronics techniques, a two-variable capacitor array and a dual-layer planar coil were integrated into a flexible ergonomic substrate, avoiding hybrid-like connections in the implantable set. The proposed arrangement (capacitor array and dual-layer coil) allows us to optimize the operation pressure range and sensing distance. The use of polyimide as both the flexible substrate and the passivation material is a key feature, ensuring a biocompatible, implantable set that is mechanically flexible and can be folded to a compact size to achieve minimally invasive implantation. An external readout device has also been developed using a discrete printed circuit board (PCB) approach to support pressure measurements. The pressure responsivity of the sensor was validated to the laboratory level using a controlled pressure chamber. The results obtained show that the capacitance value of the sensor changed from 5.68 pF to 33.26 pF as the pressure varied from 0 to 300 mmHg. Correspondingly, the resonance frequency of the implantable set shifted from 12.75 MHz to 5.27 MHz. The sensitivity of the capacitive sensor was approximately 0.58 pF/mmHg and the typical response time was 220 ms. The wireless system performance was evaluated in both air and synthetic biological tissue using a Maxwell–Wien bridge circuit. The results showed a sensing distance longer than 3.5 cm, even under moderate misalignment conditions (up to 1.5 cm). The output voltage was successfully measured, ranging from 502.54 mV to 538.29 mV, throughout the full pressure range, with a measurement error of ±2.2 mV.

## 1. Introduction

The measurement of human vital signs with miniaturized sensors plays an important role in medical applications for early diagnoses, disease treatments, and preventive health services. In particular, the measuring of blood pressure (BP) is an important biomarker to gain insight into several hemodynamics parameters, such as stroke volume, heart rate, and cardiac output, which are closely related to cardiovascular diseases (CVDs) [1,2,3]. Since hypertension is an important risk factor in the development of diseases, such as strokes, heart failure (HF), aneurysm, and chronic renal failure [1,2,3,4], the monitoring of BP can effectively reduce the incidence of these CVD-related diseases.

Currently, the cuff-based sphygmomanometer is the most commonly used device to measure static BP in the general population [5,6]. However, BP measurements obtained with this device are relatively noisy [7,8]; this variability can be due to several factors, such as human error, environmental factors, patient factors, device calibration, cuff size, and the placement of the device. In contrast, continuous or ambulatory BP monitoring provides an accurate and real-time monitoring method for better predicting and controlling hypertension-related diseases [2,3,9].

In clinical practice, intra-arterial measurement is the gold standard for continuous BP monitoring [2,9,10]. This method is a form of invasive monitoring and is performed by the cannulation of a peripheral artery, which increases the risk of infection, inflammation, and bleeding in the patient. In addition, it is overly invasive for routine inspection and daily monitoring [3]. In recent years, the rapid development of implantable devices has made it possible to perform the continuous monitoring of some physiological parameters. Thus, the development of implantable sensors for continuous and real time BP monitoring has attracted significant research attention. Depending on the method of supplying power, these types of sensors can be classified as either active or passive. The active devices include a power system for their operation, usually a battery, while the passive devices do not require an internal power source; these devices are usually integrated with inductor coils that wirelessly obtain the electric power needed for their operation [2,9]. By avoiding the use of an integrated battery, the structure of a passive sensor is simplified, is easier to miniaturize, and offers an almost unlimited device lifetime [2,9,11].

Recently, many implantable wireless sensors have been proposed for continuous BP monitoring [2,12,13,14,15,16,17,18,19]. Among them, the CardioMEMS HF System [17,18], an implantable sensor that measures pulmonary arterial pressure (PAP), is currently the only FDA-approved device for remote hemodynamic monitoring. Multiple studies have shown that CardioMEMS can reduce the risk of HF hospitalization by 37% [20]. Similarly, the Cordella PAP Sensor [19] is an implantable hemodynamic system for measuring PAP; however, this prototype is not currently approved for clinical use. In both cases, the sensor includes a variable capacitor and an inductor that form an LC resonator. The micromachining capacitor can be used to sense pressure changes, and the shift in the resonant frequency of the LC resonator can be wirelessly retrieved by an external readout unit.

These systems and most of the reported pressure sensors are generally designed to be implanted into the pulmonary artery (PA), as the pressure in this location can be associated with a number of diseases, such as HF, pulmonary hypertension, and aortic aneurysms [9]. In addition, implanting pressure sensors into the PA offers a number of design and manufacture advantages, such as a reduced pressure range (0 to 80 mmHg), larger devices, and less-invasive surgical approaches. However, the pressure measured in the PA cannot be directly related to variations in the cardiac cycle, which plays a key role in BP measurements. Therefore, if a reliable ventricular pressure sensor can be fabricated and implanted, it could open up new diagnostic and therapeutic possibilities because the left ventricle, LV, is the chamber of the heart responsible for pumping oxygenated blood to the circulatory system [3,16,21]. The real complexity for accomplishing this proposal is high, but the potential for new research in cardiovascular care is the aim. As is known, the continuous monitoring of the LV pressure not only can effectively reduce the risk of CVD-related diseases, such as strokes, HF, aortic aneurysms, chronic renal failure, and coronary heart disease, to name a few, but also significantly improve the management and treatment of these conditions, offering hope for better patient outcomes.

In this work, we report the design, fabrication, and validation of a new, flexible, wireless pressure sensor for the continuous, full-range monitoring of ventricular pressure. The proposed design is supported by our previous work, reported in [9,11], where we demonstrated that an array of two touch-mode, capacitive pressure sensors (TMCPS), arranged in parallel and connected with a double-layer coil, allowed us to obtain a high-efficiency, passive, wireless pressure sensor (92% at a readout distance of 3.5 cm). This sensor also featured a reassuringly wide operating range (5 to 300 mmHg), setting it apart from the existing sensors. Importantly, to the best of our knowledge, no single capacitive sensor has been identified that can detect the full 5–300 mmHg range, making our sensor a major advancement in the field.

The proposed sensor was fabricated considering a thin-film, monolithic approach, based on the combination of two fabrication technologies: surface micromachining and flexible electronics. The capacitive and inductive structures were defined on a single flexible ergonomic substrate, avoiding hybrid-like interconnections. This approach enhanced both the overall performance and the reliability of the system. In contrast to other works that integrate gold structures into parileno C substrates, we proposed aluminum as the structural material and polyimide, a biocompatible polymer material, as the flexible substrate and passivation material. In this way, it was possible to reduce the fabrication cost and obtain a biocompatible, efficient, and mechanically flexible device; see our previous work [22]. In addition, the incorporation of materials that are widely used in the microelectronics industry allowed us to develop a reproducible and low-temperature fabrication process.

The fabricated wireless sensor was evaluated to the laboratory level using a controlled pressure chamber and a tissue phantom as the electromagnetic medium. The obtained results demonstrate the ability to fabricate a system with a sensitivity of 0.58 pF/mmHg for a pressure range from 0 to 300 mmHg, for sensing distances greater than 3.5 cm, even under moderate misalignment conditions. The favorable achieved performance positions the proposed wireless sensor as a strong candidate for future applications in ventricular pressure monitoring.

## 2. Materials and Methods

### 2.1. System Design and Measurement Principle

**Figure 1:**
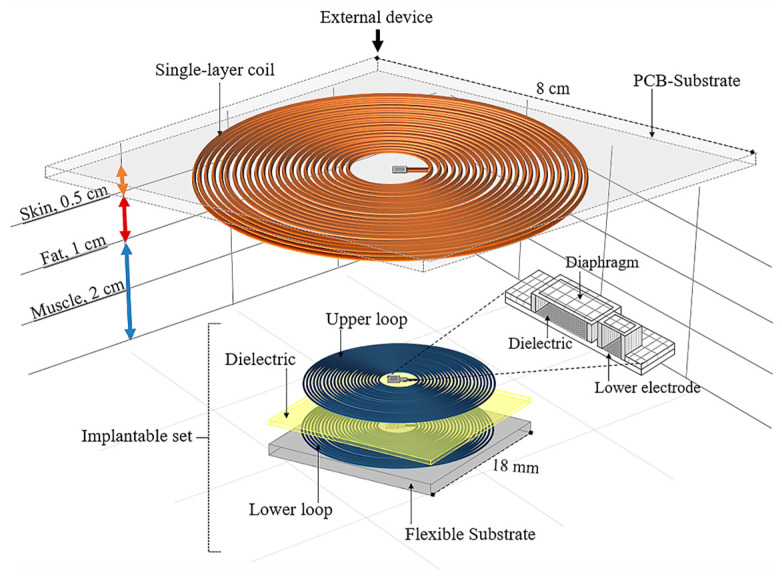
Schematic diagram of proposed wireless pressure sensor.

The proposed wireless pressure system is designed to have the ability to monitor ventricle pressure in a full-range, continuous mode. Figure 1 depicts the related schematic design. The system consists of two functional parts: an implantable set and an external readout device. The implantable set, designed to be placed inside the left ventricle at a depth of 3.5 cm from the outer surface of the body, and the external device, positioned outside of the body, in close contact with the chest, work together to ensure the system’s adaptability. To achieve a reliable wireless readout performance and a full operating pressure range, the implantable set integrates two touch-mode, capacitive pressure sensors (TMCPS), combined with a dual-layer planar coil.

The proposed planar coil is formed by two superimposed aluminum coils, connected in series and electrically insulated by a polyimide dielectric layer (1.5 µm-thick). Each coil contains 28 loops of 160 µm width, with internal and external radii of 1 mm and 18 mm, respectively. The thickness of the lower coil was set to 2 µm, while the upper coil was set to 1 µm. This selection of the thickness values allows for the improvement of the quality factor (Qs) of the implantable coil and permits the adequate deformation of the diaphragm of the two-capacitor array. The dual-layer coil maximizes space efficiency and enhances the coupled inductance, which, in turn, improves the system’s sensitivity.

The array of two TMCPS was mechanically designed to achieve a dynamic and full pressure range of 5–300 mmHg [9,10,11]. In order to cover this wide pressure range, two capacitive structures compose the integrated scheme to operate in different pressure ranges; one capacitor (555-µm side) is mechanically designed in order to respond under the lower LV pressure regime (diastolic period), and the second capacitor (300-µm side) is designed to get a response under the highest LV pressure regime (systolic period).

**Figure 2:**
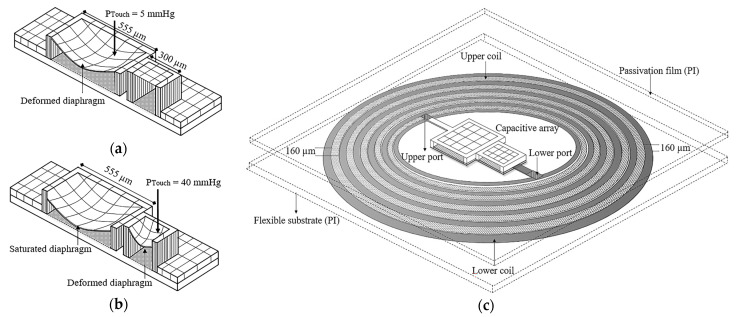
Schematic diagram of the implantable set: (**a**,**b**) cross sections of the capacitor array and its expected behavior and (**c**) the electrical connection between the two-capacitor array with the dual-layer coil.

Figure 2a,b illustrates the two-capacitor array and its expected behavior. According to the drawing, the large capacitor has an initial operating pressure (P_touch_) of 5 mmHg, while the small one operates from 40 mmHg onwards. Thus, the device can cover the full diastolic–systolic pressure range of the LV. To create a reliable resonant circuit for communication with the external device, the two capacitors are arranged in parallel and are electrically connected to the dual-layer coil; see Figure 2c. The two-capacitor array is fabricated by using the same materials and fabrication procedure as for the coils, to facilitate integration. A bilayer of polyimide (PI) and silicon oxide (SiO_2_) constitutes the dielectric material in this case. The thickness is maintained almost constantly for the components, as well; that is, for the lower electrode and the diaphragm, we use aluminum (Al) as the structural material, with thicknesses of 1 µm and 2 µm, respectively.

The external readout device allows flexibility regarding materials and structural dimensions. The design consists of a single-layer coil, connected to a variable discrete capacitor. The design parameters are as follows: several turns (27), width (160 µm), thickness (1 µm and 2 µm), self-inductance (L = 20.05 µH), and outer diameter (8 cm). Therefore, the two RCL circuits resonate at the same operating frequency (13.56 MHz). For its fabrications, the readout coil design considers the use of a discrete printed circuit board (PCB) approach; the PCB design integrates ports for the electrical connection of some discrete components, a power supply, and a signal register.

**Figure 3:**
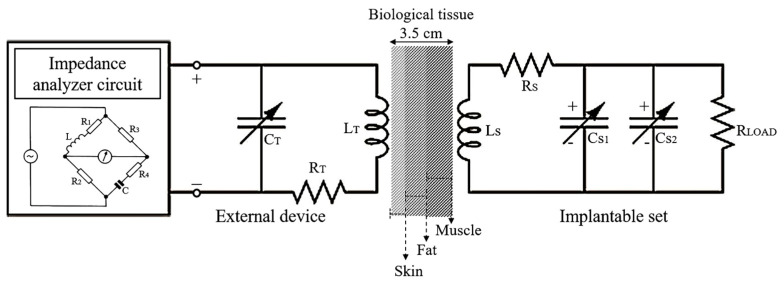
Passive electrical sensing schematic of the ventricular pressure system.

Figure 3 shows the passive electrical sensing scheme of the proposed wireless, ventricular pressure sensor. Based on the corresponding implantable LCR circuit, Equation (1) allows us to calculate the resonance frequency for an ideal sensor (f_s_), where L_s_ and C_s_ represent the sensor’s inductance and capacitance, respectively:

(1) $$f_{s}=\frac{1}{2\pi \sqrt{L_{s}C_{s}}},$$

As can be seen from Equation (1), the resonance frequency of the implantable set depends on the capacitance, which depends on blood pressure fluctuations. Therefore, blood pressure variation can generate a proportional signal in real-time by measuring the resonance frequency variation. In this case, the variation of the equivalent capacitance (C) is the sum of the individual capacitances of the two-capacitor array (C_s1_ and C_s2_) at a given pressure. The value of individual capacitance at no pressure is equal to

(2) $$C=\frac{\varepsilon_{0}\varepsilon_{r}A}{d},$$

where ε_0_ represents the permittivity of free space, ε_r_ is the relative permittivity of the dielectric material of the capacitor, A represents the contact area of the two plates, and d is the separation distance between the two plates.

The input impedance viewed from the external coil can be expressed as [23]

(3) $$Z_{\mathrm{in}}=R_{T}+j2\pi fL_{T}\left[1-{\left(\frac{f_{T}}{f}\right)}^{2}{+k}^{2}\frac{{\left(f/f_{s}\right)}^{2}}{1-{\left(f/f_{s}\right)}^{2}+j\frac{R_{s}}{\sqrt{L_{s}/C_{s}}}\frac{f}{f_{s}}}\right]$$

where R_T_, L_T_, and f_T_ are the resistance, inductance, and resonance frequency, $f_{T}=1/\left(2\pi \sqrt{L_{T}C_{T}}\right)$, of the reader coil, respectively; f is the excitation sweep frequency and k is the coupling factor, $k=a^{2}b^{2}/\sqrt{\mathrm{ab}{\left(a^{2}+X^{2}\right)}^{3}}$; where a and b are the outer radii of the external and the implantable coils, respectively; and X is a readout distance.

When the implantable set is excited at the resonant frequency (f = f_s_), the impedance of the RCL circuit becomes purely resistive. Thus, the input impedance of the external coil, coupled with the implantable sensor, can be written as

(4) $$Z_{\mathrm{in}}=R_{T}+2\pi f_{s}L_{T}k^{2}Q_{S}+j2\pi f_{s}L_{T}\left[1-{\left(\frac{f_{T}}{f_{s}}\right)}^{2}\right]$$

with $Q_{S}=\sqrt{L_{s}{/C}_{s}}/R_{s}$ being the quality factor of the sensor, at resonance. Further, when the external coil frequency resonance is selected to be smaller than the sensor resonance frequency (and assuming that R_T_ is much smaller than the coupled resistance), Equation (4) simplifies to

(5) $$Z_{in}=2\pi f_{s}L_{T}\left(k^{2}Q_{S}+j\right)$$

When the sensor’s capacitance changes, Equation (3) shows that the impedance phase shifts to either lower or higher frequency values, which can be registered by a readout electronic module. A distinguishable phase dip of the input impedance is desirable for a more favorable performance of the sensor’s wireless readout [2].

As shown by Equation (3), for Z_in_, the coupling term (the last term in the equation) can be enhanced by boosting k, which can be achieved by any of the following: (1) maintaining full alignment (angularly and transversally) between the implantable set and the external coil; (2) optimizing the parameters affecting k; (3) decreasing R_s_; or (4) increasing L_s_. This work aimed to pursue the last two options, minimize R_s_ and increase L_s_, through the use of a thick metal layer and the configuration of a dual layer for the sensor coil (lower coil).

### 2.2. Impedance Simulation

The previous results of the simulation of the LRC system were reported in [9,11]; they served us to verify the feasibility of the detection of pressure changes by considering the input impedance of a coupled external coil. In that work, CoventorWare^®^ MEMS and COMSOL Multiphysics 5.6 were the tools used to evaluate the electromagnetic and mechanical behavior of the wireless sensor. The obtained parameters were the range of frequency for the implantable set (13.56 MHz to 5.23 MHz for an applied pressure range of 5 mmHg to 300 mmHg), a quality factor (Q_s_) of 11.5, and a coupling factor (k) of 0.054. These values were applied in this work to evaluate the input impedance change and its corresponding phase magnitude. In this case, MATLAB R2024a was used for the computations.

**Figure 4:**
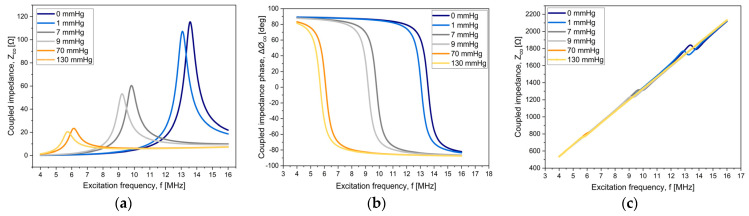

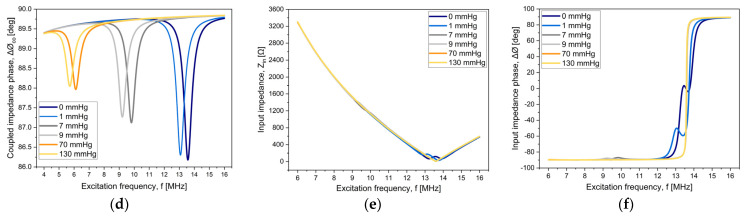
The input impedance of the LRC system: magnitude and phase. Coupled term of Equation (3): (**a**,**b**); condition at the resonance frequency of the external coil, smaller than the sensor resonance frequency: (**c**,**d**); and the full expression of the input impedance: (**e**,**f**).

Figure 4 shows the magnitude of the input impedance and that of the corresponding phase. Firstly, in parts (a) and (b), only the coupled term is shown as a function of the excitation frequency, for six different values of pressure (or equivalently, six values of resonance values). As observed, the magnitude of the coupled impedance diminishes as the sensor resonance frequency departs from the resonance at null pressure. For the phase, frequency displacements of the phase transition (variations from 90° to −90°) are observed, and they coincide with their respective resonance frequencies.

The resulting plots of when the resonance frequency of the external coil is smaller than the sensor resonance frequency are presented in Figure 4c,d. The phase shows the typical phase dip for these types of systems [23,24]. Finally, in parts (e) and (f), we show the resulting plots for the proposed system. In this case, for frequencies located away from the resonance frequency at null pressure, the influence of the external coil overrides the sensor response, reducing the measuring range of the system, particularly when the performance is via measuring the magnitude of the impedance. As noted in our previous work [9,11], the measurement of the pressure is through the measurement of the resonance frequency.

Overall, the presented simulation complements the support for the feasibility of the proposed wireless sensor’s readout modality.

### 2.3. Fabrication Process

**Figure 5:**
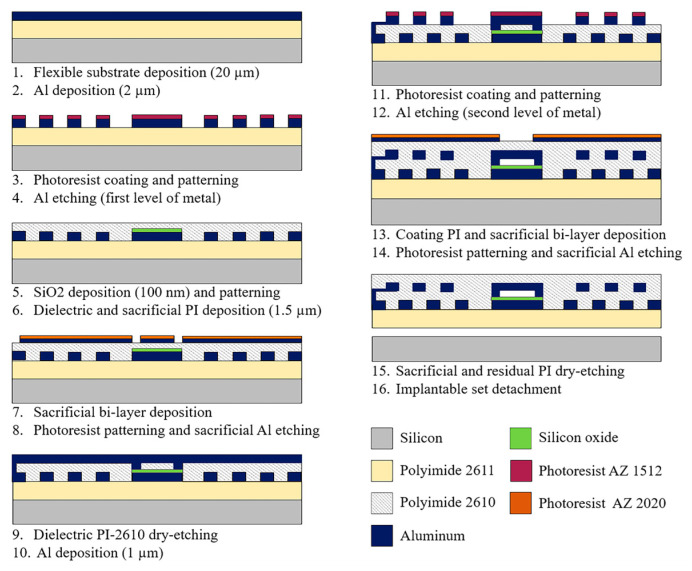
Schematic diagram of the implantable set fabrication process.

The implantable set was fabricated considering a thin-film, monolithic approach, based on the combination of two fabrication technologies: surface micromachining technology and flexible electronics. This approach enabled us to conform the capacitive and inductive structures into a single flexible ergonomic substrate, avoiding hybrid-like connections. Figure 5 shows the fabrication process flow diagram for the implantable set. The process was started by defining a 20 µm-thick polymer layer as the flexible substrate. Polyimide (PI) 2611 (HD MicroSystems™, Parlin, NJ, USA) was chosen as the substrate material because of its biocompatibility, mechanical flexibility, and ease for adjusting the film thickness. Furthermore, our previous work substantiated the selection of polyimide, in which an experimental LC prototype was successfully implanted beneath the conjunctiva of a rabbit’s eye. The polyimide-based sensor effectively conformed to the curvature of the cornea and demonstrated excellent biological stability. The sensor remained implanted for six months; during this time, it exhibited no signs of rejection. Tissue irritation disappeared within three weeks, and the animal showed no evidence of immune or toxic reactions [22].

The PI-2611 was spun at 2000 rpm using a WS-650-BT spin coater (Laurell Technologies Corporation, Lansdale, PA, USA) and cured on a hot plate MODEL HS40A (Torrey pines scientific, Carlsbad, CA, USA) at 380 °C for two hours. The polymer deposition was repeated twice, consecutively, to achieve the required thickness of the substrate (20 µm). In addition to this, after each deposit, the PI surface was treated with oxygen plasma for 30 s using a microRIE Technic series 800 system (UCLA NanoLab, Los Angeles, CA, USA); this treatment increases the surface roughness of the polyimide, which improves the adhesion between the polymer films and a subsequent metal layer. The properties of polyimide, including mechanical flexibility (as a function of thickness), biocompatibility, and durability, were optimized in our previous work, presented in [22,25].

A first structural aluminum (Al) (Kurt J. Lesker Company^®^, Jefferson Hills, PA, USA) layer of 2 µm thickness was deposited on the PI substrate, using an e-beam evaporation process. Then, a layer of AZ-1512 photoresist (Microchemical GmnH, Ulm, Germany) of 1.5 µm thickness was spin-coated and baked on a hot plate at 90 °C for 60 s. A standard photolithography process was performed on the Al film using the MA/BA GEN4 PRO series mask aligner (Süss MicroTec, Garching, Germany) and the AZ 726 MIF developer (MicroChemicals GmnH, Ulm, Germany); the selective etching of the structural Al proceeded in a solution containing phosphoric acid (H_3_PO_4_) (ACS reagent, ≥95% in H_2_O, Merck KGaA, Darmstadt, Germany), glacial acetic acid (CH_3_OOH) (glacial, reagent plus^®^, ≥99%, Merck KGaA, Darmstadt, Germany), and nitric acid (HNO_3_) (ACS reagent 70%, Merck KGaA, Darmstadt, Germany) in a volume ratio of 25:7:1 (Al-etch solution). Next, the photoresist layer was dissolved in acetone (acetone ≥ 99.9%, Merck KGaA, Darmstadt, Germany) for 10 min in an ultrasonic cleaner (Elma Schmidbauer GmbH, Singen, Germany).

Following, a dielectric bilayer composed of silicon oxide (SiO_2_) and PI-2610 (HD MicroSystems™, Parlin, NJ, USA) was deposited. The SiO_2_ layer of 100 nm thickness was deposited through a plasma-enhanced chemical vapor deposition technique (RF-PECVD, MVSystem LCC, Denver, CO, USA), and it was selectively etched with tetrafluoromethane (CF_4_) plasma over 10 min, using the microRIE system. The SiO_2_ layer was defined on the lower electrode to isolate the metal plates of the two-capacitor array electrically. Following, the PI-2610 was spun at 3000 rpm and then cured on a hot plate at 380 °C. The PI-2610 layer also serves as a sacrificial material to form a cavity between the two-capacitor plates. PI-2610 was selected because it allows for a thinner film thickness (1 µm to 2.5 µm) than polyimide 2611 (3.5 µm to 9 µm). Then, a sacrificial Al layer was deposited, 100 nm-thick, and a 1.2 µm-thick AZ 2020 photoresist (Microchemical GmnH, Ulm, Germany) layer was deposited. Then, a standard photolithography process was realized. The AZ-2020 photoresist patterns were developed by using the AZ 326 MIF developer (MicroChemicals, GmnH, Ulm, Germany). The sacrificial Al layer was selectively etched using an Al-etch solution.

**Figure 6:**
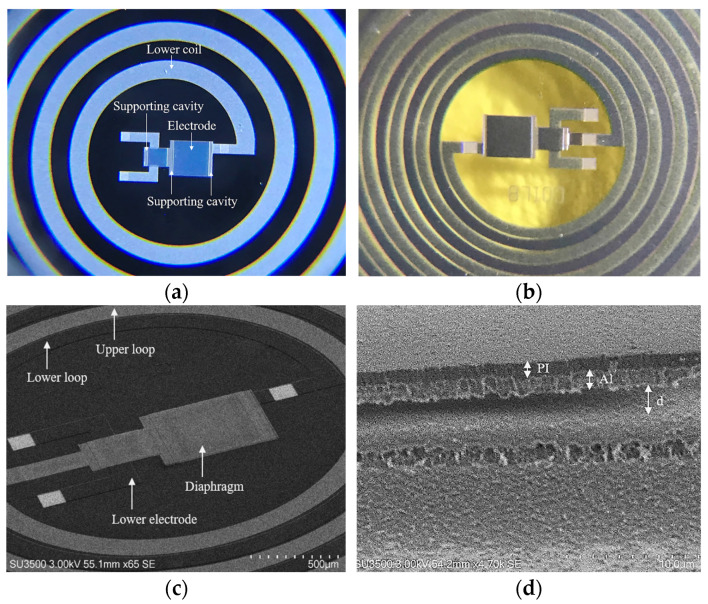
Results of the fabrication process: (**a**) the diaphragm anchor support cavities defined through the sacrificial PI, (**b**) the implantable set after the release process, (**c**) the top-view SEM image of the capacitive array and its connection with the dual-layer coil and (**d**) the cross-sectional SEM image of the capacitive sensor.

Next, the PI-2610 was selectively etched for 15 min by using oxygen plasma to form the contact between the lower and upper coils and the diaphragm anchor support cavities (see Figure 6a). Subsequently, a second layer of structural Al of 1 µm thickness was deposited and selectively etched, following the methodology previously described for the first layer of structural Al. At this step, the upper coil and the diaphragm array were defined. After that, an additional PI-2610 layer was deposited on the whole device to ensure both electrical insulation and biocompatibility. PI was chosen as the passivation material due to its compatibility with the flexible substrate, enabling the effective encapsulation of the implantable assembly. Its mechanical flexibility, biocompatibility, and high corrosion resistance make it an ideal choice for this application.

Then, a sacrificial Al layer was deposited and selectively etched to form an orifice for removing the sacrificial layer and covering the final shape of the implantable set. The residual and sacrificial PI were removed by oxygen plasma for eight consecutive hours using the microRIE system. The etching of the sacrificial polyimide is a critical step in the fabrication process because the long processing time can cause the polyimide to overheat. To overcome this issue, shorter etching cycles (carried out every 30 min) were implemented to prevent excessive heat buildup.

Finally, the polyimide-based sensor was detached from the silicon wafer in DI water, see Figure 6b. The fabricated device was analyzed using scanning electron microscopy (SEM) model JSM-7800F (JEOL, Peabody, MA, USA); Figure 6c shows the top-view SEM image of the capacitive array and its connection with the dual-layer coil; Figure 6d shows the cross-sectional SEM image of the capacitive sensor, where it is possible to observe (i) the separation distance between the metal plates of a capacitor without sacrificial material and (ii) a composed diaphragm of Al/PI: Al being the structural material and PI the coating material of the device.

### 2.4. Wireless Pressure Sensor Characterization Methods

**Figure 7:**
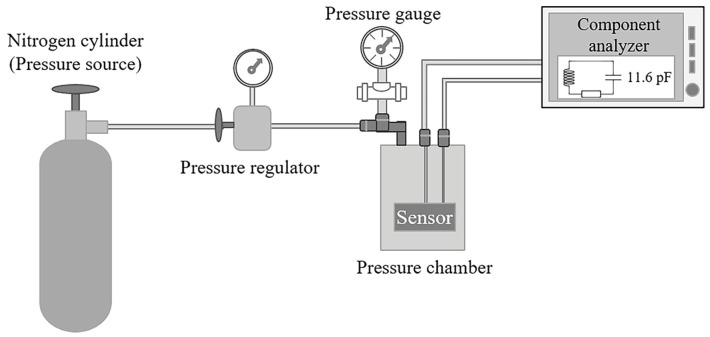
Experimental setup for measuring the capacitive change in the sensor.

When external pressure is applied to the diaphragm of the two-capacitor array, it undergoes deformation, which changes the capacitance value. This change in the capacitance shifts the resonant frequency of the implantable set. The capacitive response of the sensor as a function of the applied pressure is presented. Figure 7 shows the experimental setup for measuring the pressure response of the capacitor array. As can be seen, a commercial volumetric pressure regulator model HV-100 (HEISE, East Berlin, CT, USA) and a digital pressure gauge DP4000 series (Omega Engineering, San Pedro Garza García, Mexico) are part of the pressure control of the sensor, with a 0.05 mmHg tuning resolution. The sensor was placed in an in-house pressure chamber at room temperature.

The capacitance change was registered for an applied pressure range from 0 to 300 mmHg in 1 mmHg steps using a Wayne Kerr RLC meter model 6520A (Altamonte Springs, FL, USA). The pressure source was nitrogen gas, and the change in pressure was manually regulated.

**Figure 8:**
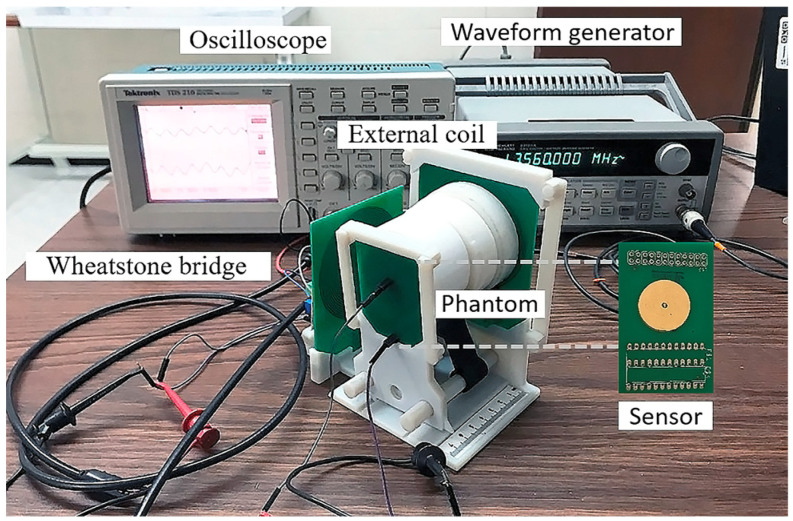
Experimental setup to measure the wireless response of the pressure sensor.

Wireless sensor characterization assumes the near-field electromagnetic coupling between the implantable set and the external RCL device (readout coil). A printed circuit board (PCB) process was used to fabricate the readout coil, see Figure 1. Additionally, a variant of the Wheatstone bridge circuit (Maxwell–Wien bridge) was integrated into the external RCL circuit to measure the output impedance at a specific frequency, expressed in terms of the voltage. Figure 8 shows the experimental setup for measuring the wireless response of the implantable set.

The dual-layer coil was mounted on a stage to control the degree of misalignment concerning the axis of the readout coil. The values of L_1_ and R_1_ of the bridge circuit are equal to those of the readout coil. When the external pressure is zero, C_1_, R_2_, and R_3_ can be varied, so as to balance the bridge circuit, resulting in a null voltage between points B and C. As the pressure is varied from zero, the capacitance value is modified and the resonant frequency, fs, shifts accordingly. Changes in the fs modify Z_in_, which, in turn, is reflected as a variation in the voltage signal between the B and C of the bridge circuit.

A sine-wave signal, Hewlett Packard 33120a (Keysight Technologies, Santa Rosa, CA, USA), supplies the bridge circuit with a sine-wave signal of 13.56 MHz. The induced voltage for the implantable set and at the output of the bridge circuit’s output is measured through an oscilloscope, Tektronix TDS 210. The distance between the readout coil and the sensor coil, X, was varied from 0 cm to 8 cm, in steps of 0.5 cm. Air and synthetic biological tissue (phantom model) are also used as an electromagnetic core. Considering that once the implantable set is placed inside the LV, the blood flow may alter the alignment between the coils, an analysis of this condition was done: a misalignment between the axes of the readout coil and sensor coil was set to take values from 0.5 cm to 2.5 cm, in steps of 0.5 cm. The impedances of the readout and sensor coils were measured by a probe station Summit 1200 and the Wayne Kerr RLC meter.

## 3. Results

### 3.1. Fabricated Implantable Set

**Figure 9:**
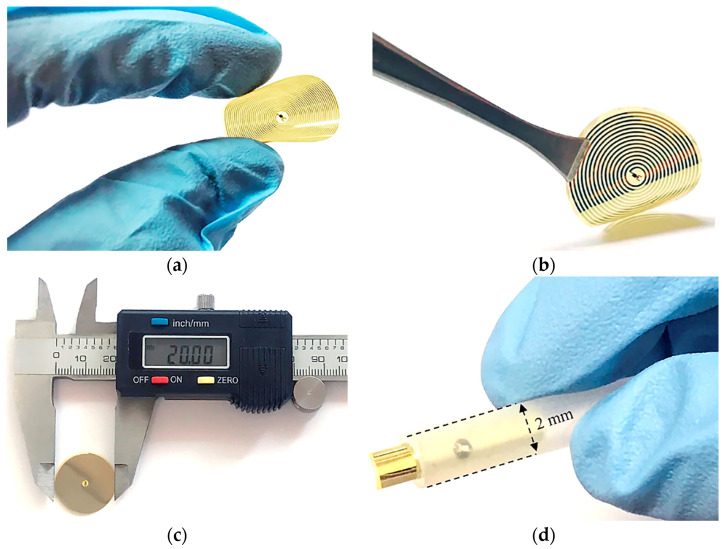
Fabricated implantable set. (**a**,**b**) Size features, (**c**) the size of the full implantable set, and (**d**) the implantable sensor inserted into a PE tube.

Figure 9 shows the fabricated implantable set. Figure 9a,b shows the fabricated two-capacitor array and dual-layer coil structures; Figure 9c,d shows the dimensional features of the complete device (20 mm in diameter). Figure 9d illustrates the implantable set’s insertion into a 2.3 mm diameter PE tube, which resembles a typical catheter used in the cardiac catheterization insertion technique; this indicates that the implantable set is compatible with catheter-based delivery methods.

We obtained a mechanically flexible, robust, miniaturized implantable set. In addition, the incorporation of materials that are widely used in the microelectronics industry allowed us to develop a reproducible, low-cost, low-temperature fabrication process.

### 3.2. Pressure Sensor

**Figure 10:**
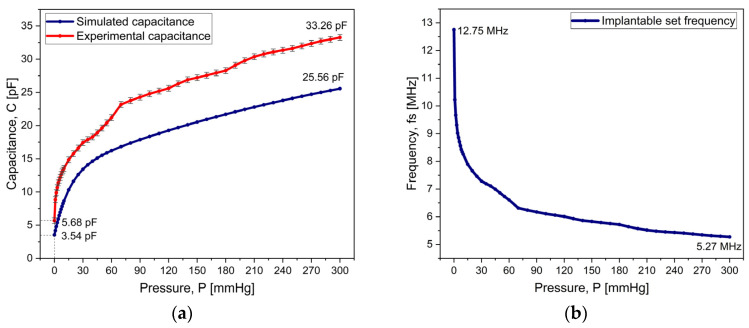
Experimental results for the responsivity of the implantable set: (**a**) capacitance (from numerical simulations and from the experiment) versus applied pressure and (**b**) resonant frequency.

Figure 10a shows the results corresponding to the sensor’s responsivity, where the capacitance changes detected by the sensor are shown for a range of applied pressure from 0 to 300 mmHg. The resulting capacitance values are between 5.68 pF and 33. 26 pF. The figure shows the simulated and experimental measurements, and differences between them can be observed: for the lower-pressure part, approximately 2 pF, and for the maximum pressure, approximately 7 pF. This discrepancy is mainly due to the wiring between the sensor and the measuring device and to deviations in the structural parameters occurring during manufacturing.

Concerning the pressure characterization, the average responsivity of the capacitive sensor is 0.58 pF/mmHg; this value is computed by taking the average of the local derivative of the capacitance–pressure curve, given by

(6) $$R_{\lambda }={\left(\Delta C/\Delta P\right)}_{P},$$

∆P is a certain change in pressure and ∆C is the corresponding change in capacitance, at a certain pressure value, P. The obtained value of R_λ_ implies that the proposed sensor can present a relatively high responsivity.

In Figure 10a, at each pressure value, the capacitance was measured 10 times with a 5-min interval between measurements. The precision of the measurements (indicated by the error bars) is evaluated as 1.48% (with a confidence level of 68%) by using the relative percentage standard uncertainty, $e=\sqrt{\sum_{m=1}^{N}{\left(C_{m}-\bar{C}\right)}^{2}/(N-1)}/\bar{C}\times 100$, where C_m_ is the m-th measurement of capacitance, $\overset{-}{C}$ is the average value, and N is the number of measurements [7]. This relatively low value of uncertainty indicates the high sensitivity of the sensor. Recalling that the responsivity of the system is 0.58 pF/mmHg, this error value corresponds to about ±8.6 mmHg, at the maximum pressure, which is the worst case. This pressure uncertainty is within the accepted standard values in the medical community.

If the simulation value for fs at zero pressure is considered to be 13.56 MHz, a variation of about 8% is observed. This variation is largely due to the expected variability that occurs during the manufacturing process. However, these variations do not affect the resulting performance of the sensor.

By using Equation (1) and the data in Figure 10a, we calculated the shift in the resonant frequency of the implantable set as a function of the applied pressure—this is presented in Figure 10b. At zero pressure, fs is measured as 12.75 MHz; this reference value decreases as the pressure increases, reaching a value of 5.27 MHz.

**Figure 11:**
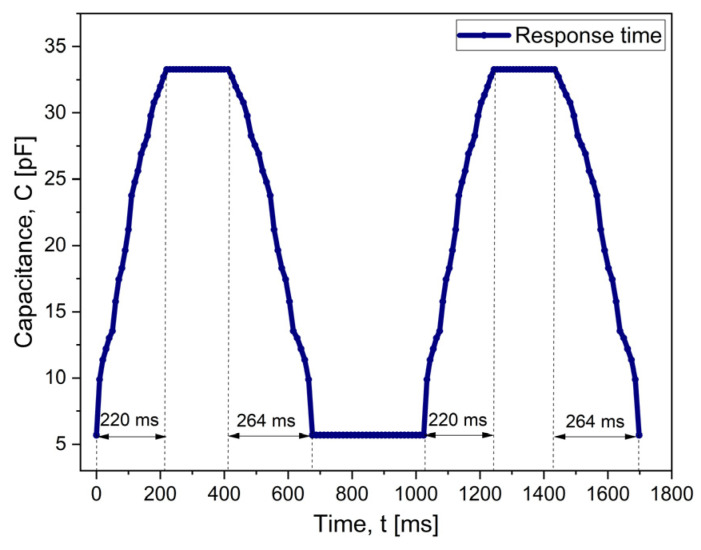
The evaluation of the performance of the two-capacitor array: response time.

Figure 11 shows the performance of the two-capacitor array in terms of response time. The implantable sensor has a response time of 220 ms when the applied pressure is increased from 0 to 300 mmHg. When the system is set back, i.e., from 300 mmHg to 0 mmHg, the array reaches its base value of capacitance in 264 ms, as shown in Figure 11; overall, these response time values indicate that the sensor can be used for continuous real-time monitoring.

### 3.3. Wireless System

Before realizing the coupling between the dual-layer coil and the external coil, the self-inductance of each coil was measured as 27.4 µH and 21.83 µH, respectively. These values can be compared with those obtained from the simulation, Ls = 39.93 µH and L_T_ = 21.29 µH. The marked mismatch for the inductance values of the implantable coil stems from intrinsic structural variations observed during the fabrication process.

By selecting the values for Ls and Cs—see Figure 10a for the behavior of this latter parameter—the resonant frequency, fs, is tuned to the design value of 13.56 MHz. The magnetic coupling between the implantable coil and the external device was analyzed as follows. Firstly, the axes of both the external coil and the implantable coil were aligned to coincide. Applying a 13.56 MHz sine-wave signal with a peak-to-peak voltage of 5 V to the Maxwell–Wien bridge circuit, the induced voltage on the implantable coil, Vin, was subsequently measured. This procedure uses two magnetic cores: air and synthetic biologic tissue (phantom model). A misalignment arising from lateral displacement between coils was analyzed as well. Under these conditions, V_in_ was measured as a function of the separation distance between the coils, which spanned a range of 0.5 cm to 8 cm in increments of 0.5 cm.

**Figure 12:**
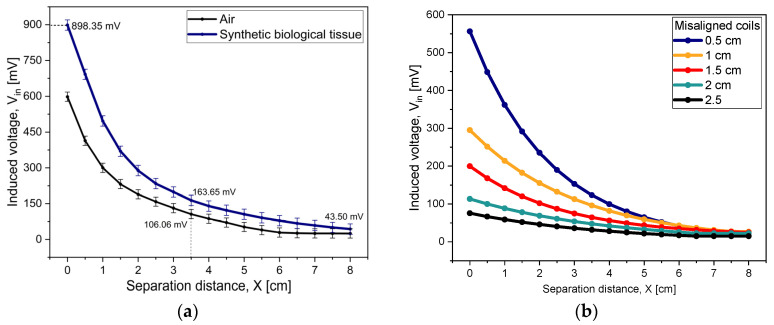
Induced voltage on the implantable coil, as a function of the separation distance between the coils. (**a**) With correct alignment, for two magnetic cores: air and synthetic biological tissue. (**b**) With misalignment, for synthetic biological tissue.

Figure 12 shows the results of this analysis. In (a), the effect of the magnetic core is included (no misalignment between the coils is assumed): the biological tissue outperforms air (differences in the impedance matching between the magnetic cores and the coils may be involved [26]); the registered voltage is around 1.5 times higher for the biological tissue. At a separation distance of 3.5 cm, which is a practical value, the induced voltage is sufficiently large to induce a voltage on the external device. The induced voltage rapidly decays for larger separation distances, setting a useful limit to about 6 cm.

Realizing prescribed lateral displacements between the implantable and the external coils, the resulting induced voltage performs as shown in Figure 12b (the magnetic core corresponds to the synthetic biological tissue). As expected, V_in_ decreases as the coupling factor decreases. As noticed, the system can accommodate misalignments of up to 2.5 cm, as the voltage signal is greater than the noise signal, at a separation distance of 3.5 cm. However, this inductive power transfer (IPT) system is limited to misalignments smaller than 1.5 cm. Since the LV chamber of the heart sets a limit to the maximum lateral length, of 1.16 cm [27], the pressure sensor can be implanted and operated in this chamber. In Figure 12a, the error bars indicate an average error of 3% for the induced voltage, which amounts to ±1.3 mV in the worst case (at X = 8 cm), and it is of the order of the resolution of the oscilloscope that was used for the measurements. In Figure 12b, we do not show the error bars, since they are the same as those presented in Figure 12a.

**Figure 13:**
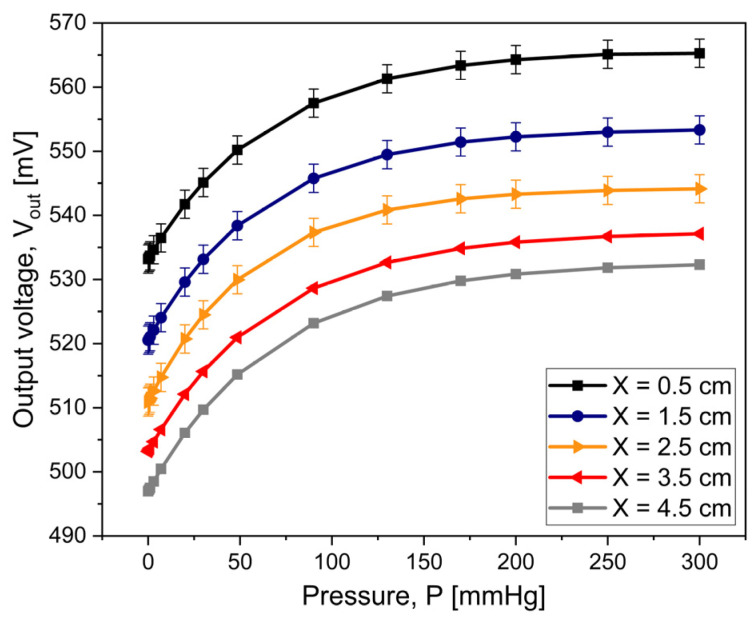
Output voltage versus pressure applied to the implantable set.

When operating the wireless system, the measurable variable corresponds to the output voltage, V_out_, at the terminals A and B of the bridge circuit of the external device. Figure 13 presents the resulting output voltage when varying the applied pressure from 0 to 300 mmHg. Each curve indicates the influence of the separation distance between the coils on the output voltage. In this case, the magnetic core corresponds to the biological tissue. The supplied signal to the bridge circuit is the same as the one used when analyzing the implantable coil.

In general, Figure 13 demonstrates that is possible to establish a relationship between the pressure in the biological medium and an electrical variable that the reading device can be measured; for example, for the specific distance of 3.5 cm (red curve), V_out_ increases from 502.54 mV to 538.29 mV, for the full pressure range. Also, we can observe that the larger the separation distance between the coils, X, the smaller the output signal. Besides, Figure 13 shows that the slope of the curves is close to zero for pressure values greater than 300 mmHg, which ultimately limits the device’s useful range. In these results, the observed error was about ±2.2 mV (for displaying purposes, the error bars are scaled up). The resulting uncertainty stems mainly from the wiring of the bridge circuit.

Considering these results, the fabricated sensor can establish a range of pressure of 5 mmHg to 300 mmHg when conventional measuring equipment is taken into account. The performance of the bridge circuit allows for the system’s high sensitivity. If we selected the impedance as the measurable variable, as shown in the simulation Section 2.2, the system’s response would be limited to about 130 mmHg.

## 4. Discussion

In the designing stage, the selection of the double-layer geometry of the coil permitted the optimization of the quality factor by incrementing the length of the coil in a compact area; when the length of the coil was increased, the resistance and the self-inductance increased proportionally; since the quality factor was $Q_{S}=\sqrt{L_{s}{/C}_{s}}/R_{s}$, this parameter was reduced in this condition; then, the thickness of the coils had to be enlarged to compensate for this reduction. Due to the deposition technique’s limitations, the lower coil’s selected thickness was 2 µm; also, relatively thick layers could cause inhomogeneities. A trade-off was met between Q and the mechanical performance of the system. Then, the thickness of the upper coil was prescribed to 1 µm to ensure the adequate deformation of the diaphragm array.

The electrical wiring and vacuum hardware were key factors in the experimental setup for characterizing the sensor’s responsivity. A hermetic chamber filled with nitrogen operates at the ambient temperature. When we considered different surrounding media, in practical terms, the device’s performance was unmodified, as the permeability of nitrogen and of several types of tissues was almost the same as 1.0. Similarly, the wireless effectivity may be assumed to be unrelated to the surrounding medium as well, since the magnetic induction depends on the permeability. Conversely, the lateral misalignment between the implantable and external coils considerably affected the system’s PTE. In this case, the signal degradation may be upgraded using multiple-layer coils and larger planar coils, which ultimately improve the system’s Q.

It is important to note that the measurement capability of the system was limited by the uncertainty in the voltage output; when we used direct impedance measurements, the range of measured pressure was reduced to approximately 130 mmHg, instead of approximately 300 mmHg, by using the current bridge circuit. This impedance factor limitation emphasizes the need for future work to optimize the bridge circuit.

The development of the wireless pressure sensor holds potential for applications beyond the current scope. It can be applied to monitor intraocular and intracranial pressure, among other bodily functions. However, to broaden its applicability, the critical work of analyzing the size of the full sensor with respect to the pressure and available space for implantation remains open. This size and pressure relationship can open new applications by exploring other geometries and operational electromagnetic frequencies. While this represents a significant challenge, it also opens up promising future research and development possibilities.

## 5. Conclusions

A flexible wireless capacitive pressure sensor for full-range, continuous ventricular pressure monitoring was fabricated using surface micromachining and flexible electronics techniques. The sensor was designed with a focus on simplicity and efficiency, using only three materials: aluminum (Al) for its conductivity, polyimide (PI) for its flexibility, and silicon dioxide (SiO_2_) for its insulating properties. The novel implantable, set on a flexible, ergonomic substrate, proposes a two-variable capacitor array and a dual-layer planar coil without hybrid-like connections. The two-capacitor array enables the coverage of the wide LV pressure range, while the dual-layer coil improves the system’s power transfer efficiency (PTE) in a reduced physical area.

A practical operating range of 5 to 300 mmHg was demonstrated, with a response time of 220 ms and an approximate sensitivity of 0.58 pF/mmHg. The effect of the distance separation between the external coil and the implanted coil was evaluated using two magnetic core materials: air and synthetic biological tissue. The synthetic tissue core demonstrated superior performance compared to the air core. At a separation distance of 3.5 cm (practical value for application), a voltage of 163.65 mV was induced in the implantable coil, providing sufficient power to operate the sensor. Additionally, an analysis of the lateral misalignment between coils showed us that moderate values of this parameter, approximately 1.5 cm, are permitted.

The measurements showed an average error of ±2.2 mV, primarily attributed to the implemented electronic interface (Maxwell–Wien bridge circuit). Future efforts should focus on optimizing this interface to enhance performance. Furthermore, this sensor can also be adapted to monitor pressure in different organs, such as the aorta, pulmonary artery, eye, and brain. However, it will be necessary to reduce the coil size for the latter two applications.

## Data Availability

The data that support the findings of this study are available from the corresponding author upon request.

## Abbreviations

Acronym	Abbreviations
PCB	printed circuit board
BP	blood pressure
CVD	cardiovascular disease
HF	heart failure
PAP	pulmonary arterial pressure
FDA	Food and drug administration
PA	pulmonary arterial
LV	left ventricle
TMCPS	touch-mode, capacitive pressure sensors
Q_s_	quality factor
PTE	power transfer efficiency
PI	polyimide
SiO_2_	silicon oxide
Al	aluminum
RCL	Resistor, capacitor, and inductor
fs	Resonance frequency of implantable set
Ls	Inductance of implantable coil
Cs	Capacitance of the sensor
ε_0_	Permittivity of free space
ε_r_	Relative permittivity
R_T_	Resistance of reader coil
L_T_	Inductance of reader coil
f_T_	Resonant frequency of external device
k	Coupling factor
a	Outer radio of the external coil
b	Outer radio of the implantable coil
X	Readout distance
f	Resonant frequency
Z_in_	Input impedance
PI-2611	Polyimide 2611
RIE	Reactive-ion etching
H_3_PO_4_	Phosphoric acid
CH_3_OOH	Glacial acetic acid
HNO_3_	Nitric acid
PECVD	Plasma-enhanced chemical vapor deposition
CF_4_	Tetrafluoromethane
DI	Deionized water
SEM	Scanning electron microscopy
PE	Polyethylene
ΔP	Change in pressure
ΔC	Change in capacitance
P	Pressure
R_λ_	Responsivity
e	Relative percentage standard uncertainty
Cm	m-th measurement of capacitance
$\overset{-}{C}$	Average value of capacitance
N	Number of measurements
Vin	Induced Voltage
IPT	Inductive power transfer
Vout	Output voltage
